# Retraction: Characteristics of patients undergoing percutaneous coronary intervention for chronically total occluded arteries: a single-center observational study in India

**DOI:** 10.1097/CP9.0000000000000082

**Published:** 2024-05-03

*Cardiology Plus* Editorial Office

The Editors and Publisher retract the article “Characteristics of patients undergoing percutaneous coronary intervention for chronically total occluded arteries: a single-center observational study in India” by Khadtare *et al.* published in *Cardiology Plus* (ahead of print). A statistical error is present in Figure 1’s data.
